# A Framework for the Study of Complex mHealth Interventions in Diverse Cultural Settings

**DOI:** 10.2196/mhealth.7044

**Published:** 2017-04-20

**Authors:** Marion A Maar, Karen Yeates, Nancy Perkins, Lisa Boesch, Diane Hua-Stewart, Peter Liu, Jessica Sleeth, Sheldon W Tobe

**Affiliations:** ^1^ Faculty of Medicine Northern Ontario School of Medicine Laurentian University Sudbury, ON Canada; ^2^ Department of Medicine Queens University Kingston, ON Canada; ^3^ Department of Medicine Sunnybrook Health Sciences Centre, Sunnybrook Research Institute University of Toronto Toronto, ON Canada; ^4^ Department of Research Northern Ontario School of Medicine Sudbury, ON Canada; ^5^ University of Ottawa Heart Institute Ottawa, ON Canada

**Keywords:** mobile health, health care texting, SMS, protocol, process evaluation, process assessment (health care), health services, Indigenous, Tanzania, community-based participatory research, DREAM-GLOBAL

## Abstract

**Background:**

To facilitate decision-making capacity between options of care under real-life service conditions, clinical trials must be pragmatic to evaluate mobile health (mHealth) interventions under the variable conditions of health care settings with a wide range of participants. The mHealth interventions require changes in the behavior of patients and providers, creating considerable complexity and ambiguity related to causal chains. Process evaluations of the implementation are necessary to shed light on the range of unanticipated effects an intervention may have, what the active ingredients in everyday practice are, how they exert their effect, and how these may vary among recipients or between sites.

**Objective:**

Building on the CONSORT-EHEALTH (Consolidated Standards of Reporting Trials of Electronic and Mobile HEalth Applications and onLine TeleHealth) statement and participatory evaluation theory, we present a framework for the process evaluations for mHealth interventions in multiple cultural settings. We also describe the application of this evaluation framework to the implementation of DREAM-GLOBAL (Diagnosing hypertension—Engaging Action and Management in Getting Lower BP in Indigenous and LMIC [low- and middle-income countries]), a pragmatic randomized controlled trial (RCT), and mHealth intervention designed to improve hypertension management in low-resource environments. We describe the evaluation questions and the data collection processes developed by us.

**Methods:**

Our literature review revealed that there is a significant knowledge gap related to the development of a process evaluation framework for mHealth interventions. We used community-based participatory research (CBPR) methods and formative research data to develop a process evaluation framework nested within a pragmatic RCT.

**Results:**

Four human organizational levels of participants impacted by the mHealth intervention were identified that included patients, providers, community and organizations actors, and health systems and settings. These four levels represent evaluation domains and became the core focus of the evaluation. In addition, primary implementation themes to explore in each of the domains were identified as follows: (1) the major active components of the intervention, (2) technology of the intervention, (3) cultural congruence, (4) task shifting, and (5) unintended consequences. Using the four organizational domains and their interaction with primary implementation themes, we developed detailed evaluation research questions and identified the data or information sources to best answer our questions.

**Conclusions:**

Using DREAM-GLOBAL to illustrate our approach, we succeeded in developing an uncomplicated process evaluation framework for mHealth interventions that provide key information to stakeholders, which can optimize implementation of a pragmatic trial as well as inform scale up. The human organizational level domains used to focus the primary implementation themes in the DREAM-GLOBAL process evaluation framework are sufficiently supported in our research, and the literature and can serve as a valuable tool for other mHealth process evaluations.

**Trial Registration:**

ClinicalTrials.gov NCT02111226; https://clinicaltrials.gov/ct2/show/NCT02111226 (Archived by WebCite at http://www.webcitation.org/6oxfHXege)

## Introduction

### Pragmatic RCTs and mHealth Interventions

Explanatory randomized controlled trials (RCTs) are the gold standard for measuring efficacy, the cause and effect relationship in treatment intervention research in medicine, and are instrumental in providing evidence for Clinical Practice Guidelines (CPGs). A key requirement of explanatory RCTs is the stringent standardization of the tested intervention, which (while challenging) is realistic; for example, in pharmaceutical trials that occur in clinical settings where a single variable can be reasonably well controlled. Standardization in explanatory RCTs removes bias and thus results in substantial rigor and strong internal validity; however, in contrast, external validity (ie, the applicability in diverse clinical settings) is often compromised and consequently applying RCT results to populations outside the study’s scope can be problematic [1,2].

Health care interventions are typically difficult to standardize because they are complex with long, nonlinear implementation chains that may have unexpected outcomes in disparate settings. Similarly, mobile health (mHealth) interventions usually contain multiple active components that may require changes in the behavior of patients and providers. These behaviors in turn are driven by numerous social, cultural, and environmental factors, thus creating considerable complexity and even ambiguity related to causal chains [3,4]. Therefore, a shortcoming of the robust explanatory RCT methodology is that this approach often falls short of providing answers needed in mHealth interventions and everyday clinical care practice [5]. To facilitate the decision-making capacity between options of care under real-life service conditions, clinical trials must be pragmatic to evaluate an intervention under the variable conditions of health care settings with a wide range of participants [6].

### Evaluating and Reporting Pragmatic RCTs of mHealth Interventions

To be truly useful for decision making, pragmatic trials require detailed and standardized reporting with sufficient transparency to communicate what works and what does not work for whom and under what circumstances. The prevailing international standard for reporting trials in general is the CONSORT statement [7], which has been expanded by Zwarenstein and colleagues [6] to reflect the complexity of issues that may impact on pragmatic trials. For Web-based and mHealth interventions, Eysenbach and colleagues have called for further expansion of reporting requirements. They developed the CONSORT-EHEALTH (Consolidated Standards of Reporting Trials of Electronic and Mobile HEalth Applications and onLine TeleHealth) statement to ensure reporting of sufficient detail for replication and theory building specifically to eHealth [8]. The reporting standards are designed to provide important guidance for reporting trials in general, but they do not distinguish specific elements that should be researched during the process of implementation and juxtaposed with outcome research at the end of the trial.

Although the key research outcome of a complex intervention focuses on the effectiveness of the intervention in everyday practice, the process evaluation in contrast assesses how closely the intervention was actually implemented as intended in the study protocol. Process evaluation data explain why a study unexpectedly did not achieve anticipated outcomes or if achieved outcomes are truly a consequence of the intervention. Process evaluations therefore help to distinguish between the reasons for lack of outcome as follows: (1) a consequence of either implementation failure or (2) the failure of the intervention itself. In multisetting studies, the process evaluation also facilitates an examination of differences in outcome and documents program adaptations at various sites. Process evaluations shed light on the range of unanticipated effects an intervention may have, what the active ingredients in everyday practice are, how they exert their effect, and how these may vary among recipients or between sites [9].

### Investigating Implementation Factors in Multisetting RCTs

Although standardized reporting of pragmatic trials is crucial to our understanding of how and why complex interventions work, a difficulty related to good reporting is that neither all the active components nor implementation barriers are known before the start of a trial; instead issues are often discovered during the implementation process and may vary between settings, thus requiring an emergent rather than predetermined study of implementation. Consequently, a key opportunity for the discovery and description of the unanticipated active components is the process evaluation. We argue that standardized reporting frameworks for pragmatic RCTs must allow for flexibility to permit investigators to explore emergent processes, once work on the trial has commenced, to ensure rigorous documentation of the evolving and capricious aspects of implementation. Therefore, a process evaluation plan that systematically seeks to document not only the standardized implementation items but also the emergent aspects of implementation must be a critical piece in the documentation of pragmatic trials, particularly when diverse cultural settings are involved.

Recommendations for the design of process evaluations of complex interventions have been published [9-12]. We maintain, however, that little work has focused on developing theoretical approaches that would help researchers to reduce the complexity of the process evaluation by focusing on those functional components that are most relevant to community stakeholders as well as researchers in mHealth interventions.

Although there can be no single definition of what elements comprise the process evaluation of the implementation of mHealth interventions, we deconstruct the functional components that are most significant to researchers, policy makers and implementation science in this study. Building on the CONSORT-EHEALTH statement and participatory evaluation theory, we present a framework for the process evaluations for mHealth interventions focusing on active components of the intervention in multiple cultural settings. We also describe the application of this evaluation framework to the implementation of DREAM-GLOBAL (Diagnosing hypertension—Engaging Action and Management in Getting Lower BP in Indigenous and LMIC [low- and middle- income countries]), a pragmatic RCT and mHealth intervention designed to improve hypertension management in low-resource environments. In addition, we describe data collection tools and processes that were developed to collect the process evaluation data necessary to inform implementation of the pragmatic RCT and to inform future scale up of DREAM-GLOBAL in various geographic and cultural settings.

## Methods

### The DREAM-GLOBAL RCT

DREAM-GLOBAL is a pragmatic RCT that can be described as a complex mHealth intervention. It is a research project designed to increase the capacity for affordable, evidence-based, guidelines-driven hypertension management interventions at the patient, provider, and community level by leveraging existing mobile telecommunication technology and task shifting (findings by Yeates K et al, unpublished data, 2016). This study was funded by The Canadian Institutes of Health Research, Grand Challenges Canada, and by the Global Alliance for Chronic Diseases and involves diverse cultural and geographic settings, including Indigenous communities in Canada and the Kilimanjaro region of Tanzania.

The DREAM-GLOBAL mobile technology consisted of health care short messaging services (SMS) texting to support patient hypertension self-management and to facilitate decision support for health care providers. The program theory was informed by the Canadian Hypertension Education Program CPGs drawing on evidence for the prevention of high Blood Pressure (BP) through dietary sodium restriction, BP measurement, education interventions for health care providers and patients, inter-professional care, health systems, and interventions such as automated reminder systems [13,14].

Specifically, the DREAM-GLOBAL RCT was designed to test the effectiveness of mobile phone–based SMS feedback to patients and providers tailored to patients’ BP measurements using artificial intelligence. Briefly, in this intervention, nonmedical workers used Bluetooth-enabled BP monitors to record and transmit BP readings from patients with hypertension. Mobile phones were used for storing and transmitting the patient roster and BP readings to a server. The server then sent text messages to the participating patients with their individual results. Study participants were randomized into two groups: one group received a set of cardiovascular health promotion messages and the second group received the same set of messages and additional “active” messages that support self-management by indicating requirement for attendance at medical appointments, rationale for drug therapy, and adherence to medication regimes, and messages directly reflective of their blood pressure readings. In addition, DREAM-GLOBAL closed the loop for sharing of clinical health information among health care providers.

### The DREAM-GLOBAL Constructivist Approach to the Process Evaluation

Our review of the RCT reporting and process evaluation literature revealed that there is a significant knowledge gap related to the development of a process evaluation framework that can accommodate the values of community-based participatory research (CBPR) and the importance of acknowledging diverse cultural perspectives and settings. We used a medical anthropology approach to develop a framework reflective of participatory evaluation theory and specifically focused on meeting the information needs of academic and community stakeholders for mHealth interventions in diverse settings. The process evaluation framework was informed by the following 3 main factors:

1. A review of current process evaluation theories applied to complex interventions and established RCT reporting requirements;
2. Formative DREAM-GLOBAL CBPR in diverse cultural settings, including ethnographic notes, research engagement and implementation research notes, and reflective discussion sessions with research and community teams collected during formative research. This data represents a substantial set of qualitative data collected over the period of 1.5 years [15,16];
3. mHealth-specific topics within the context of international, culturally, and geographically diverse low-resource settings [17].

### Current Process Evaluation Theories

Various approaches to the evaluation of complex interventions have been described in the literature. Bamberger and colleagues stressed that no single methodology can address all dimensions of complexity and that a careful mapping of complexity dimensions is necessary to design an evaluation [18,19]. They offered a conceptual framework of 5 sources of complexity in the evaluation of interventions as follows: (1) the nature of the program (what does the intervention look like), (2) the context within which the program is embedded, (3) the interactions among the different stakeholders and agencies involved in the program,(4) the nature of processes of change and causality (how does the program effect change in society and how can change be captured), and (5) the nature of the evaluation process (how to deal with divergent stakeholder interests and incentives, data availability, resources, etc) [20].

In contrast, Damschroder and coworkers conducted a review of the literature and identified 5 major domains for the evaluation of implementation: intervention characteristics, outer setting, inner setting, characteristics of the individuals involved, and the process of implementation. The respective domains are further broken down into a total of 37 subcategories such as cost, policies, implementation climate, stage of change, and engagement [21].

Realist evaluation approaches have also been applied to process evaluations of complex interventions [22]. The realist evaluation questions include: “What works, for whom, in what respects, to what extent, in what contexts, and how?” To answer these questions, realist evaluators tried to identify the underlying mechanisms that explain how the outcomes were caused and the influence of context on the intervention [23]. Although the main realist evaluation questions can be adapted to various projects, there is a lack of consensus and consistency in defining the major domains of “mechanism” and “context.” This limited its utility in the development of a concise framework for process evaluations aimed at mHealth interventions, particularly when sources of complexity include culturally and geographically diverse low-resource sites, such as the DREAM-GLOBAL trial [24].

The Medical Research Council’s (MRC) guidance report is a framework for process evaluation of complex interventions and builds on the 2008 MRC guidance document for complex interventions, which recognized the importance of process evaluation within trials [11,12]. It builds on three concepts for a process evaluation design namely the description of the intervention, the mechanisms, and the context. These concepts align with realist evaluation theory, although the authors do not recommend specific evaluation theories or approaches. The authors argue for clear descriptions of intervention theory and identification of key implementation process questions and tailoring of the evaluation to the intervention.

While all these frameworks will result in the documentation of important aspects of a process evaluation, there are also serious limitations. For example, although these approaches advocate to examine the setting of the intervention, it is not clear which aspects of the setting evaluators should be focused on. Another limitation specifically associated with realist evaluation approaches is that realist philosophy presupposes that “reality can be experienced and shared by everyone in precisely the same way” [25]. However, in a multicultural context, there is often little knowledge and experience that is shared between the researchers and those who interact with the intervention. It is precisely the lack of a shared culture that makes implementation particularly difficult to predict, unless the differences in how the intervention is experienced in various cultural settings are acknowledged and actively researched. We argue that it is particularly important when dealing with evaluations in multiple cultural settings to use a constructivist approach which acknowledges the knowledge and experience constructed by the individuals and societies. Participatory evaluation theory provides an excellent framework, as this approach incorporates the perspective and lived experience of local stakeholder in all aspects of evaluation. The participatory evaluation approach includes a community-based discovery process of implementation issues that integrates local knowledge and is essential to the development of a good evaluation framework for health interventions designed to work in diverse settings.

We argue that given the complexity of mHealth interventions and the multiple cultural settings of trials such as DREAM-GLOBAL, it would be naive for an evaluator to identify a priori the most important active components of the intervention and related evaluation research questions. Local knowledge and expertise of community stakeholders is urgently needed not only for successful implementation of DREAM-GLOBAL but also to understand implementation barriers and required adaptations. We therefore intentionally moved away from notions of the detached objectivity of a positivistic oriented evaluation which would marginalize local knowledge and emergent understandings. Instead of applying a constructivist evaluation approach, the research team entered the development of the process evaluation as “learners, not claiming to know preordinately what is salient” [26]. The collaborative process, characteristic of constructivist inquiry supported a discovery process informed by dialogue, negotiation, and verification with stakeholders to identify the most relevant evaluation domains related to implementation [26].

### Formative Community-Based Participatory Research

CBPR has been defined as “a collaborative research approach that is designed to ensure and establish structures for participation by communities affected by the issue being studied, representatives of organizations, and researchers in all aspects of the research process to improve health and well-being through taking action, including social change” [27]. CBPR principles have been incorporated into DREAM-GLOBAL. During formative CBPR research for this project, we developed an information gathering and dialogue tool, consisting of the following 3 phases: (1) a community profile tool, (2) an interview guide to facilitate the discussion of strategic topics related to implementation with key stakeholders in the community, and (3) a focus group guide to lead a dialogue on community-specific issues related to the intervention. The methodology has been previously published [15]. A thematic analysis of this qualitative data as well as participant observation data from community visits and team meetings provided initial process evaluation domains and evaluation research questions. We then further compared and contrasted our formative data with the evaluation literature, specifically the MRC guidelines [12] and CONSORT-EHEALTH extension. We ensured that the basic process evaluation domains would address the most important research questions that are meaningful and of immediate importance to community and policy maker stakeholders.

The finalization of the process evaluation domains was facilitated by monthly team discussions and several evaluation meetings over the period of 12 months where the literature, formative research evidence, ethnographic notes, and stakeholder discussion notes were analyzed, and eventually consensus was reached on the most significant process evaluation domains.

### Integration of the Process Evaluation Research Into the DREAM-GLOBAL Trial

**Figure 1:**
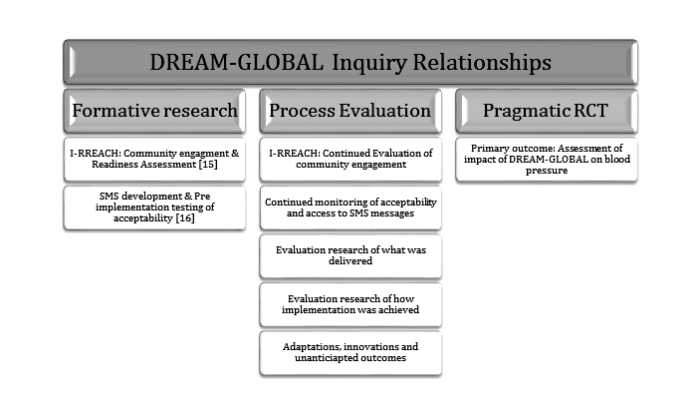
DREAM-GLOBAL process evaluation relationship to formative research and trial research (I-RREACH, Intervention and Research Readiness Engagement and Assessment of Community Health Care).

The pragmatic trial is designed to test the program theory of DREAM-GLOBAL—to facilitate the control of BP in low-resource environments through evidence-based text messaging, targeting patients, and enabling feedback to health care providers. The formative research was designed to generate technological, cultural, social, and health systems data to determine feasibility and, if necessary, plan for tailoring the implementation for each DREAM-GLOBAL site [15,16]. The process evaluation protocol was nested within the pragmatic trial and followed the formative research before implementation of the trial and preceded outcome research (see Figure 1). The purpose of the process evaluation was primarily to evaluate the process of implementation of the trial; however, it will also generate information that will be valuable for potential posttrial scale up of DREAM-GLOBAL. The objectives of the process evaluation were to examine (1) what was delivered, including the quality (fidelity) and quantity (dose); (2) how delivery of the intervention was achieved; as well as (3) how the mHealth intervention required adaptations or innovations within the context of international, culturally, and geographically diverse low-resource settings [28] and 4) any unanticipated outcomes [12].

### Ethics

The study involved Indigenous people and is built foremost on strong commitment to respectful, CBPR with First Nations communities as partners in research as outlined in the Tri-Council Policy Statement (TCPS 2) [29]. We therefore sought academic ethics review as well as community-based First Nations ethics review for the clinical trial as well as the process evaluation research. Furthermore, the research team also sought formal approval and permission for DREAM-GLOBAL implementation from First Nations decision makers in each of the participating communities after local, in-person presentations. Academic ethics approvals include the following: (1) Queen’s University Health Sciences and Affiliated Teaching Hospitals Research Ethics Board, Kingston, Ontario (DMED-1603-13); (2) Sunnybrook Health Sciences Centre Research Ethics Board, Toronto, Ontario, (#182-2013) and (3) the National Institute for Medical Research Tanzania (NIMR/HQ/R.8a/Vol.IX/1698). Community-based ethics review in First Nations communities included The Cree Board of Health and Social Services of James Bay, Ontario and Manitoulin Anishinaabek Research Review Committee (MARRC), Ontario. The study was also formally approved by decision-making bodies in all participating communities through Band Council Resolutions in participating First Nations.

### Trial Status

The implementation of the RCT (ClinicalTrials.gov registration: NCT02111226. 2014) is currently ongoing and is expected to be completed by December 2017. The data analysis had not begun at the time of submission of this protocol.

## Results

### Organizational Levels of the Process Evaluation

**Figure 2:**
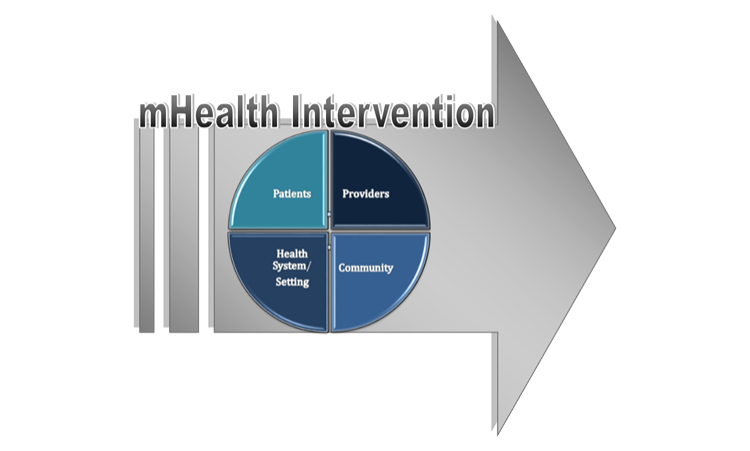
Major factors that interact with the DREAM-GLOBAL mHealth intervention.

Four human organizational levels of participants impacted by the mHealth intervention were identified in our analysis and included patients, providers, community and organizations actors, and health systems and settings (see Figure 2). These four levels correspond with the organizational evaluation domains (see Textbox 1).

These four organizational levels critically impact on the primary outcome of the mHealth intervention, that is, improvements in BP. The process evaluation framework therefore examines implementation from each of these four organizational perspectives.

**Human organizational levels.**

- Patient participants comprise the primary target population of the intervention, in this case people with hypertension are enrolled in the study.
- Provider participants are care and service providers whose work is to some degree altered by the intervention.
- Community and organization members are people whose immediate social environment impacts on the intervention, for example, those who permit the implementation or approve changes in work flow. They may also be decision makers.
- Health system and setting members are people or structures that impact on the implementation at a systems level, such as local and district level decision makers and National health policy makers.

### Primary Implementation Themes in the Process Evaluation

We thematically reanalyzed qualitative data collected during the formative research and found four primary implementation themes that interact with the four organizational domains described above. The primary implementation themes for each of the organizational domains were (1) identification of the major active component of the intervention (or program theory), (2) the technology of the intervention, (3) cultural congruence, and (4) task shifting. In addition, participant observation research and ongoing collaboration meetings with community stakeholders helped to identify emergent issues and the importance of these issues. We therefore learned the importance of documenting unintended consequences, which became our fifth primary implementation theme.

### Development of Evaluation Questions

The interaction between the organizational levels and the primary implementation themes will be explored in this process evaluation. Using the four organizational domains and their interaction with the primary implementation themes, we developed detailed evaluation research questions and the identified data or information sources that we concluded would best answer our questions. The evaluation questions and data sources are presented in Multimedia Appendix 1.

For example, one issue identified as a primary implementation theme in our formative work was the notion of task shifting. Task shifting in health care is a process where specific tasks are delegated to health care workers who have fewer qualifications but have received competency training [30]. In DREAM-GLOBAL, the task of measuring BP is shifted from a medical to a nonmedical worker. Applying this implementation theme to the four domains of our framework, we developed queries to explore the impact of task shifting on each of the four human organizational levels:

1. Patients have to be comfortable with the task shifting.
2. Nonmedical providers should be confident and willing to take on this new task, and medical providers should be confident on the nonmedical providers to accept that measurements are accurate, in order to act on them
3. The organizational decision makers have to be in a position where they can assign the new tasks.
4. Health system decision makers must support the shift instead of posing barriers (eg, funding or licensing issues).

## Discussion

### Principal Findings

The DREAM-GLOBAL RCT constitutes a complex mHealth intervention because it is designed to affect change in the behavior of patients, providers, and local systems in order to achieve the primary outcome: lowering BP through management according to CPG. The DREAM-GLOBAL RCT intervenes at multiple points in the care normally provided to patients with hypertension. It combines a chronic care management approach with medical task shifting to community health resource and community health workers in order to ensure hypertension care is delivered according to CPGs, even in low-resource environments such as Indigenous communities in Canada and Tanzania.

Given the complexity of the program, extensive formative research was necessary to understand the local health care system before initiating program implementation. This was necessary for the program to be adapted to patients and providers in diverse cultural contexts and health systems [15]. However, we also realized that some adaptations to optimize integration with existing health services and to minimize undue increased workload and systemic barriers could not be anticipated before implementation and that required an openness to flexibility during the process of implementation. In line with Bumbarger and Perkins [28], we wanted to document the unanticipated issues and distinguish between “innovation” (skilled implementers actively attempting to make an intervention better fit their population or setting) and “drift” (unintentional shortcomings arising from barriers to full implementation) [12].

The research team anticipated the possibility of emerging elements that should be examined in a process that may not have been obvious during implementation planning. The research team preplanned a discovery process through formative and CBPR.

### Applicability of the DREAM-GLOBAL Framework to Other mHealth Interventions

The DREAM-GLOBAL pragmatic trial involves several interacting components targeting changes in the patient, provider, health organizations, and health systems, which makes it a complex intervention. This is arguably true for almost all mHealth interventions, and our proposed framework based on human organizational level domains is therefore a useful and a clearly focused framework for process evaluations of complex mHealth interventions.

Our framework chart identified several primary implementation themes related to the intervention that we claim to be explored in process evaluations of mHealth interventions:

Major active components of the intervention: Craig and colleagues discussed for the importance of describing and evaluating how the intervention works by clearly describing how the active ingredients exert their effect. “Only by addressing this kind of question can we build a cumulative understanding of causal mechanisms, design more effective interventions and apply them appropriately across group and setting [10].” We believe that it is necessary to explore active components from the perspective of the four human organizational levels of patients, providers, organizations, and systems.

Technology: Technology is by definition the most integral component of any mHealth intervention and therefore requires close scrutiny in process evaluation at different organizational levels.

Cultural congruence: The importance of the significant additional complexity introduced with the inclusion of diverse cultural settings of implementation has been stressed in the literature [9,11]. With increasing cultural diversity within many regions of the world due to human migration as well as the application of interventions in various settings, a focus on the important role of cultural factors and the arising need for adaptation for cultural congruence is an important emergent area of study of mHealth interventions.

Task shifting: Task shifting is one way to expand the health work force in low-resource countries, and it is a necessary component of mHealth when the interventions require new ways for providers to interact with patients.

Unintended consequences: Many teams have underscored the need for process evaluations to capture unintended consequences and the importance of using a variety of methods to describe these in detail [10,11,31].

Clearly, the primary implementation themes of DREAM-GLOBAL are strongly supported in the literature, which underscores their relevance to process evaluations of mHealth interventions, in general. When the framework is applied to interventions other than DREAM-GLOBAL, additional primary implementation themes could be added by researchers once identified, and added to the framework chart. Evaluation questions within the framework chart should be adapted for each intervention.

### Implications for mHealth Interventions in Diverse Cultural Settings

The DREAM-GLOBAL process evaluation protocol provides a framework to document how this intervention was implemented in various Indigenous communities in Canada and rural villages in Tanzania; and if the same intervention was implemented and received in similar ways in different communities or not. Our framework has several strengths related to work in diverse cultural settings.

First, this framework allows for exploration of emergent topics and verification with multiple stakeholder perspectives instead of imposing a rigid linear framework that would have put us at risk of not identifying and analyzing key unanticipated implementation factors. The four human organizational levels (patients, providers, community and organizations, and health systems and settings) can be applied to other mHealth interventions to guide the development of the most salient evaluation research questions. Although implementation themes may vary slightly in other mHealth interventions, following our approach will help to identify the relevant implementation themes.

Second, our collaborative social constructivist I-RREACH approach allowed us to tap into the tacit knowledge of stakeholders by collecting qualitative data through field notes and extensive dialogue and negotiation. Community stakeholders and providers have tacit local knowledge which can support, strengthen and optimize the intervention in the local context; academic team members on the other hand have technical expertise to ensure that essential elements of an intervention are preserved in various settings [15].

Third, CBPR—the research approach employed for DREAM-GLOBAL—is an excellent epistemological fit with a social constructivist evaluation approach. Clearly, CBPR shares the orientation of social constructivist evaluation in that it incorporates and legitimizes multiple value systems, perspectives, and stakeholders and emphasizes the role of dialogue and negotiation and their link to action and improvements. Therefore, as part of the study implementation, the community was involved in shaping how the research would be implemented to provide the maximal benefit for the community and these processes were critically important for the successes of the study.

Fourth, the process evaluation is intended to support the succinct reporting of the DREAM-GLOBAL RCT according to the CONSORT-EHEALTH.

Finally, we found that the formative qualitative research on community engagement and the development of the SMS messages we conducted before implementation of DREAM-GLOBAL [15,16] were also useful in informing the process evaluation. The I-RREACH process was implemented in five Indigenous communities and two rural villages in Tanzania. A total of 135 informants participated in 12 focus groups and 7 interviews. After the sessions were completed, 83 informants participated in an evaluation of the I-RREACH session. During the development of culturally safe SMS messages, a total of 45 informants from 3 Indigenous communities and 1 community in Tanzania participated in four focus groups. This rich formative qualitative research provided deeper understanding of community issues related to the implementation of a hypertension mHealth intervention in our thematic analysis and confirmation for the emerging evaluation domains for the process evaluation protocol.

We acknowledge that an important factor in our approach is that it relies on a collaborative team approach which includes local community experts and a well-functioning interdisciplinary academic team.

Our research supports the notion that process evaluations of complex mHealth interventions cannot be fully designed in advance and instead should employ a framework that allows for emergent research. When an mHealth intervention is to be deployed in multiple cultural settings, our research suggests that a participatory constructivist approach to the development of the process evaluation is necessary to identify relevant evaluation questions that incorporate local knowledge and expertise.

### Conclusions

Eysenbach and colleagues stated that the CONSORT-EHEALTH statement is “only the first step and the guideline will be very much a living document in an iterative and ongoing development process” [8]. We have developed from this checklist, to identify items that are well suited to be studied during implantation of an mHealth intervention during the implementation using process evaluation methodologies. We succeeded in creating an uncomplicated approach to the development of a process evaluation framework for mHealth interventions that provide key information to stakeholders, which can optimize implementation of the pragmatic trial and can be used to inform scale up. The human organizational level domains used to focus primary implementation themes in the DREAM-GLOBAL process evaluation framework are sufficiently supported in our research and literature that they can serve as a valuable tool for other mHealth process evaluations.

## Appendix

Multimedia Appendix 1 Framework chart with evaluation research questions.

## Abbreviations

BP: blood pressure
CBPR: community-based participatory research
CONSORT-EHEALTH: Consolidated Standards of Reporting Trials of Electronic and Mobile HEalth Applications and onLine TeleHealth
CPG: clinical practice guidelines
DREAM-GLOBAL: Diagnosing hypertension—Engaging Action and Management in Getting Lower BP in Indigenous and LMIC
I-RREACH: Intervention and Research Readiness Engagement and Assessment of
LMIC: low- and middle-income countries
mHealth: mobile health
MRC: Medical Research Council
RCT: randomized controlled trial
SMS: short message service
