# Supplementary material for: A Framework for the Study of Complex mHealth Interventions in Diverse Cultural Settings
Source: JMIR Mhealth Uhealth. 2017 Apr 20;5(4):e47. doi: 10.2196/mhealth.7044 (PMC5418524; doi:10.2196/mhealth.7044)
Supplement: Multimedia Appendix 1 [file mhealth_v5i4e47_app1.pdf]

Multimedia Appendix

Framework chart with evaluation research questions.

Human organizational level domains	Primary implementation themes					Data type and tool
	**Major active components of the intervention**	**Technology of the intervention**	**Cultural congruence of the intervention**	**Task shifting enabling the intervention**	**Unintended consequences arising from the intervention**	
**Patients**	What is the intervention as received by patients? (Include queries into fidelity, dose and reach)<br><br>Have there been any changes in patient's access to health services?<br><br>Was there good recruitment and retention of patients?	Can the patient use the technology?<br><br>Are phones affordable/accessible?<br><br>Is the server/software successfully sending the correct messages?<br><br>Are cell phones user-friendly and appropriate delivery mechanism of messages?<br><br>What are the training and support requirements for patients?	Were the components of the intervention culturally appropriate?<br><br>Were the SMS messages culturally safe?<br><br>What are patients' and providers' thoughts and understanding of SMS messages?	Was the task shifting accepted by patients?	What were the unanticipated power dynamics (if any)?<br><br>What changes had to be made to the intervention to accommodate the reality of the patients?	Qualitative quantitative, I-RREACH pre-implementation and 3-month post, technology log/dashboard; ethnographic notes from regular site visits, researcher and community partner meetings
**Providers (community health workers, doctors, etc.)**	What is the intervention as received by providers?<br><br>Communication and collaboration between health staff and DG research team<br><br>Do providers manage hypertension according to CPGs?	Can the community health worker/ community health nurse use the technology?<br><br>What are the technological troubleshooting areas? What training and support was required?	Is the care provided by providers culturally safe?	What new/additional roles/tasks are required for providers?<br><br>Do providers support the task shifting?<br><br>Were non-medical staff confident to take on the new tasks?<br><br>What task had to be shifted or	What training and support is required long-term?<br><br>What were the unanticipated power dynamics (if any)?<br><br>What changes had to be made to the intervention to accommodate the reality of the providers?	Qualitative quantitative, I-RREACH pre-implementation and 3-months post, technology log/dashboard; ethnographic notes from regular site visits, researcher and community partner meetings

	Do providers enroll patients effectively?			newly implemented or newly trained?<br><br>What level of training was required to support task shifting initially and long-term?		
**Community and organizations**	What is the intervention applied to the community/organization?<br><br>Are communities and key organizations engaged in the research?<br><br>What is the quality of the engagement?<br><br>Are stakeholders aware of and promoting DREAM-GLOBAL?<br><br>Was there more awareness in the community?	Was it possible to share the field measurement health information (BP readings) with primary care providers?<br><br>Are field measures successfully integrated into medical records?<br><br>Were new relationships established as a result?	In which aspects of the project was the community involved? Which not?<br><br>How was engagement initiated and maintained?<br><br>Was the engagement culturally appropriate?	Has the task shifting supported or impeded the health care leaders and managers?<br><br>Was sufficient training provided to allow the program to be delivered by the community?	What were the unanticipated power dynamics (if any)?<br><br>What changes had to be made to the intervention to accommodate the reality of the community/organizations?	Qualitative quantitative, I-RREACH pre-implementation and 3-months post, technology log/dashboard; ethnographic notes from regular site visits, researcher and community partner meetings
**Health system/setting**	What is the intervention from the health systems/settings perspective?<br><br>Is the health system receptive to the intervention?<br><br>Are current health policies and realities congruent with the intervention?<br><br>Are medications accessible?	Is cell coverage/signal strength available/adequate for the region?	Is the health services system receiving the patients culturally competent?	Is there sufficient time in the worker's day to take on the new role?<br><br>Is the task shifting supported or impeded by policies and funding arrangements?	What unanticipated events or outcomes related to the health system affected the intervention?<br><br>How did this impact on the intervention?<br><br>What are the positive and negative consequences?<br><br>What changes had to be made	Qualitative quantitative, I-RREACH pre-implementation and 3 months post, technology log/dashboard; ethnographic notes from regular site visits, researcher and community partner meetings

	How does the intervention fit with current health services and infrastructure? (availability of clinic space, staff)				to the intervention to accommodate the reality of the health system/setting?<br><br>What is required to sustain the intervention in each country or region?	

Maar MA, Yeates K, Perkins N, Boesch L, Hua-Stewart D, Liu P, Sleeth J, Tobe SW
A Framework for the Study of Complex mHealth Interventions in Diverse Cultural Settings: The DREAM-GLOBAL Pragmatic Randomized Controlled Trial Process Evaluation Protocol
JMIR Mhealth Uhealth 2017;5(4):e47
URL: http://mhealth.jmir.org/2017/4/e47/
